# Integrated Spheroid-to-Population Framework for Evaluating PFHpA-Associated Metabolic Dysfunction and Steatotic Liver Disease

**DOI:** 10.21203/rs.3.rs-5960979/v1

**Published:** 2025-03-04

**Authors:** Brittney O. Baumert, Ana C. Maretti-Mira, Douglas I. Walker, Zhenjiang Li, Nikos Stratakis, Hongxu Wang, Yinqi Zhao, Fabian Christoph Fischer, Qiran Jia, Damaskini Valvi, Scott M. Bartell, Carmen Chen, Thomas Inge, Justin Ryder, Todd Jenkins, Stephanie Sisley, Stavra Xanthakos, David E. Kleiner, Rohit Kohli, Sarah Rock, Sandrah P. Eckel, Michele A. La Merrill, Max M. Aung, Matthew P. Salomon, Rob McConnell, Jesse Goodrich, David V. Conti, Lucy Golden-Mason, Lida Chatzi

**Affiliations:** 1 Department of Population and Public Health Sciences, Keck School of Medicine, University of Southern California, Los Angeles, CA, United States; 2 USC Research Center for Liver Diseases, Division of Gastrointestinal and Liver Diseases, Department of Medicine, Keck School of Medicine, University of Southern California, Los Angeles, CA, United States; 3 Gangarosa Department of Environmental Health, Rollins School of Public Health, 1518 Clifton Road, NE, Atlanta, GA, United States; 4 Barcelona Institute for Global Health, ISGlobal, Dr. Aiguader 88, 08003, Barcelona, Spain; 5 Department of Biomedical and Pharmaceutical Sciences, University of Rhode Island, Kingston, RI 02881, United States; 6 Department of Environmental Medicine and Climate Science, Icahn School of Medicine at Mount Sinai, New York, NY, United States; 7 Department of Environmental and Occupational Health, University of California, Irvine, Irvine, CA, United States; 8 Department of Surgery, Northwestern University Feinberg School of Medicine, Chicago, IL, United States; 9 Ann & Robert H. Lurie Children’s Hospital of Chicago, Chicago, IL, United States; 10 Cincinnati Children’s Hospital Medical Center, Department of Pediatrics, University of Cincinnati College of Medicine, Cincinnati, OH, United States; 11 Department of Pediatrics, Baylor College of Medicine, Houston, TX, United States; 12 National Institutes of Health, National Cancer Institute, Center for Cancer Research, Bethesda, MD, United States; 13 Division of Gastroenterology, Hepatology and Nutrition, Children’s Hospital Los Angeles, Los Angeles, CA, United States; 14 Department of Environmental Toxicology, University of California, Davis, CA, United States

**Keywords:** PFAS (Per- and Polyfluoroalkyl Substances), Liver Disease, Lipid Metabolism, Multi-Omic Profiles, Liver Spheroids

## Abstract

The rising prevalence of metabolic dysfunction-associated steatotic liver disease (MASLD), particularly among pediatric populations, requires identification of modifiable risk factors to control disease progression. Per- and polyfluoroalkyl substances (PFAS) have emerged as potential contributors to liver damage; however, their role in the etiology of MASLD remains underexplored. This study aimed to bridge the gap between human epidemiological data and in vitro experimental findings to elucidate the effect of perfluoroheptanoic acid (PFHpA), a short chain, unregulated PFAS congener on MASLD development. Our analysis of the Teen-LABS cohort, a national multi-site study on obese adolescents undergoing bariatric surgery, revealed that doubling of PFHpA plasma levels was associated with an 80% increase in MASLD risk (OR, 1.8; 95% CI: 1.3–2.5) based on liver biospies. To further investigate the underlying mechanisms, we used 3D human liver spheroids and single-cell transcriptomics to assess the effect of PFHpA on hepatic metabolism. Integrative analysis identified dysregulation of common pathways in both human and spheroid models, particularly those involved in innate immunity, inflammation, and lipid metabolism. We applied the latent unknown clustering with integrated data (LUCID) model to assess associations between PFHpA exposure, multiomic signatures, and MASLD risk. Our results identified a proteome profile with significantly higher odds of MASLD (OR = 7.1), whereas a distinct metabolome profile was associated with lower odds (OR = 0.51), highlighting the critical role of protein dysregulation in disease pathogenesis. A translational framework was applied to uncover the molecular mechanisms of PFAS-induced MASLD in a cohort of obese adolescents. Identifying key molecular mechanisms for PFAS-induced MASLD can guide the development of targeted prevention and treatment.

## Introduction

Metabolic dysfunction-associated steatotic liver disease (MASLD), previously known as nonalcoholic liver disease (NAFLD)^1^, refers to a spectrum of liver disorders, including metabolic dysfunction-associated steatohepatitis (MASH), formerly known as nonalcoholic steatohepatitis (NASH). A hallmark of MASLD is fat accumulation (steatosis) in the liver, due to chronic metabolic dysfunction^2^. The prevalence of MASLD in children has been growing in recent years, paralleling the rise in childhood obesity and metabolic syndrome^3,4^. MASLD is currently one of the most common chronic liver diseases in children worldwide, affecting approximately 10% of children in the general population^4^. Among children who are overweight or obese, the prevalence of MASLD is much higher, with an estimated–30–40%^3^. Diet restrictions, physical activity interventions, and FDA-approved drugs including glucagon-like peptide-1 (GLP-1) receptor agonists^5^ have been used with limited success in adolescents with MASLD^6^. This highlights the need for preventive measures such as identifying and intervening in modifiable risk factors.

Traditional risk factors for MASLD, such as excess energy intake, sedentary lifestyle, and genetics, cannot fully explain the MASLD epidemic in children^7^. Moreover, emerging evidence indicates that exposure to endocrine-disrupting chemicals can promote metabolic changes that result in fatty liver disease, a hypothesis referred to as the ‘Toxicant Fatty Liver Disease’^8–10^. Per- and polyfluorinated substances (PFAS), a large class of synthetic fluorinated organic chemicals, are ubiquitous worldwide. These chemicals have been used in industrial applications and consumer products, including water-repellent textiles, nonstick coatings, and food packaging products for over 60 years^11^. PFAS have been detected in the blood of over 99% of individuals in the United States (U.S.)^12,13^. Production of certain PFAS, such as perfluorooctane sulfonate (PFOS) and perfluorooctanoate (PFOA), was voluntarily phased out in the U.S. during the 2000s, yet their negative health effects remain a concern because of their long half-lives (1.8–6.2 years)^12,14–16^. Consequently, newer PFAS variants, known as replacements, have been introduced, featuring shorter biological half-lives, to mitigate environmental persistence^14^. However, many of these replacements lack regulations and thorough testing regarding potential health risks, particularly during crucial developmental stages.

Research has demonstrated that PFAS can accumulate in the human body with a particular predilection for the liver^17–19^. This accumulation is associated with disruptions in several hepatic functions, notably lipid metabolism^16,20–22^. A substantial body of evidence, supported by both experimental and epidemiological studies, indicates that certain PFAS are hepatotoxic in humans, with many studies specifically linking PFAS exposure to lipid dysregulation^20^. However, critical gaps in the literature remain. These include: a) whether overweight or obese individuals are more susceptible to PFAS-induced hepatotoxicity, b) the potential hepatotoxic effects of less-studied or replacement PFAS compounds, and c) identification of the specific metabolic pathways impacted by PFAS that are indicative of liver damage^16,20,22,23^.

**Figure 1.**
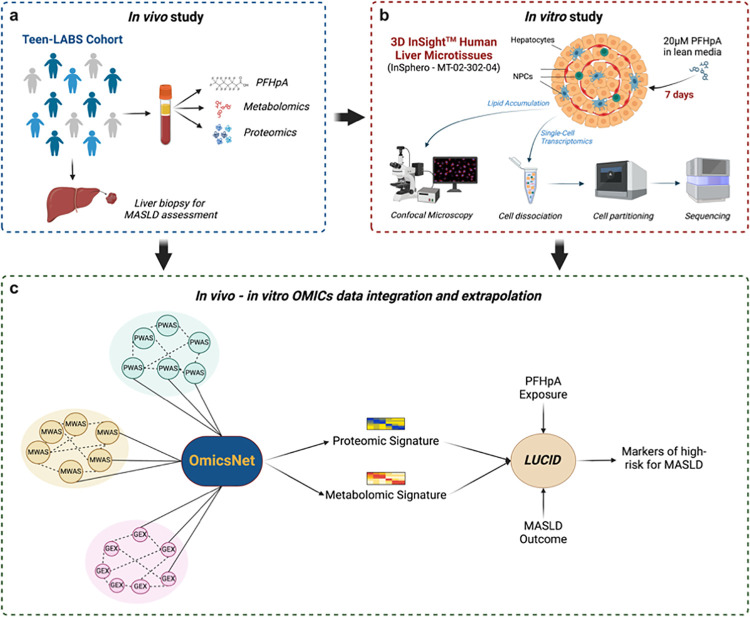
Translational framework. (A) Teen-LABS study design. (B) Human liver spheroids composed of primary hepatocytes and non-parenchymal cells (NPCs) were exposed to 20μM PFHpA for 7 days. Culture media was changed every 2–3 days to ensure constant PFHpA presence in culture media. At the end of the culture period, spheroids were dissociated into single cells, and viable cells were partitioned using the 10x Genomics platform. Single-cell gene expression libraries were sequenced using the Illumina platform. Created with BioRender.com. (C) Data integration workflow. We used the OmicsNet 2.0 platform to integrate 156 differentially expressed genes upregulated in PFHpA-exposed spheroids (GEX), 42 metabolites (MWAS), and 28 proteins (PWAS) upregulated in the plasma of MAFLD subjects (compared to non-MAFLD controls). After analyzing the overlapped pathways between GEX-MWAS and GEX-PWAS, we identified 19 metabolites and 6 proteins that will be used for further analysis. Created with BioRender.com

We propose a translational research framework designed to bridge scientific findings from both in vitro and human studies, with the goal of elucidating the role of PFAS in the risk and progression of Metabolic-Associated Steatotic Liver Disease (MASLD) (Fig. 1). Our investigation specifically focused on perfluoroheptanoic acid (PFHpA), a short-chain carboxylic PFAS compound that has been detected at elevated concentrations in the liver. The human study included in this manuscript represents a unique resource, as it involves histologically confirmed MASLD phenotypes derived from liver biopsies of adolescents with obesity. Our findings revealed a strong association between PFHpA exposure and the risk and severity of MASLD. Using an innovative approach, we assessed the impact of PFHpA on liver metabolism in vitro by employing 3D human liver spheroids coupled with single-cell transcriptomics. This methodology enabled the identification of the key metabolic pathways that were disrupted by PFHpA exposure. We then integrated multi-omic datasets from both human studies and in vitro experiments, using advanced statistical methods. This comprehensive analysis allowed us to pinpoint the specific protein and metabolite signatures associated with the development of MASLD in the context of PFHpA exposure. Our study presents a novel strategy to identify individuals at a high risk of developing PFAS-induced MASLD, paving the way for the development of early intervention strategies.

## Results

### Human Study: PFHpA increases the risk for MASLD in adolescents – Insights into Steatosis Severity and Disease Progression

**Figure 2.**
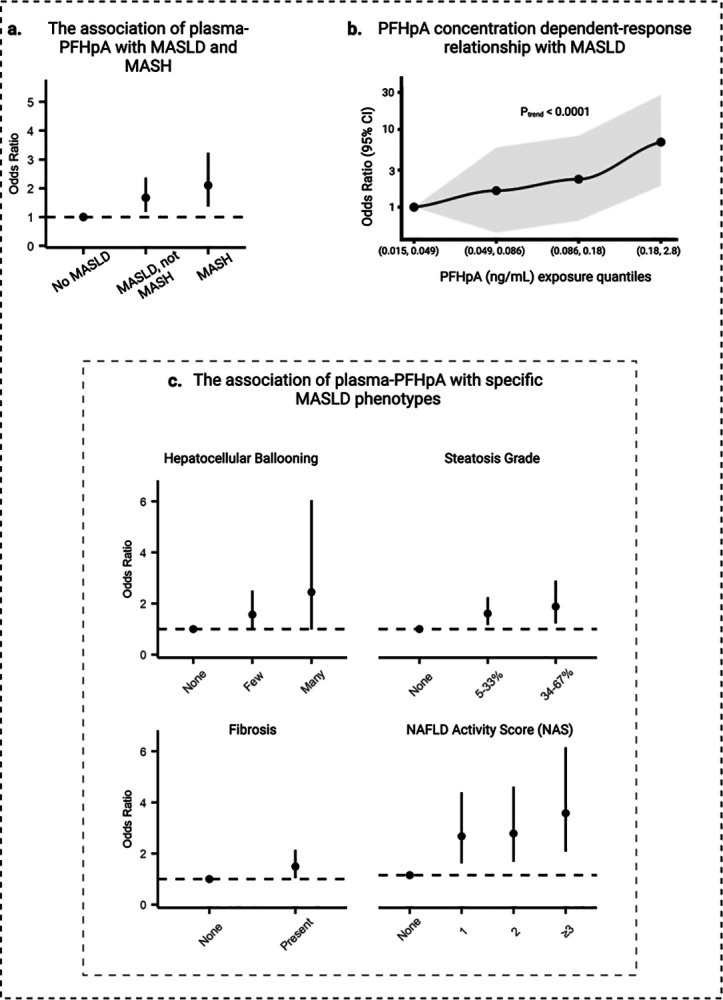
PFHpA exposure, histopathological determined outcomes of MASLD in the Teen-LABS study. (A) Shows the odds ratio (OR) and 95% confidence intervals (95% CIs) for the association between PFHpA (ng/mL) and histopathological determined MASLD (N=136). Multinomial logistic regression between PFHpA and severity of MASLD (No MASLD, MASLD not MASH, MASH). The models controls for: age, sex, race, parental income, study site. Results are shown as log2 of PFHpA exposure and therefore interpreted as per doubling of PFHpA exposure. (B) The association between quantiles of PFHpA and histopathological determined MASLD (N=136). Model adjusted for: age, sex, race, parental income, study site. Results are shown as log2 of PFHpA exposure and therefore interpreted as per doubling of PFHpA exposure. (C) The association between PFHpA and histopathological determined outcomes of SLD (N=136). Figure 1c shows the odds ratio (OR) and 95% confidence intervals (95% CIs) for the association between PFHpA (ng/mL) measured in plasma and refined outcomes of SLD. Multinomial logistic regression between PFHpA and hepatocellular ballooning, steatosis grade, and NAFLD Activity Score (NAS); Logistic regression between PFHpA and presence (y/n) of fibrosis. All models adjusted for: age, sex, race, parental income, study site. Results are shown as log2 of PFHpA exposure and therefore interpreted as per doubling of PFHpA exposure.

This study included 136 adolescents with severe obesity who underwent bariatric surgery (Table 1). Based on the liver biopsy results, participants were categorized into three groups: 55 (40%) were classified as non-metabolic associated steatotic liver disease (non-MASLD), 51 (38%) as MASLD without steatohepatitis (MASLD not MASH), and 30 (22%) as MASLD with steatohepatitis (MASH) (Table 2). Among the 8 PFAS congeners analyzed, plasma-PFHpA (mean = 0.13 ng/mL, SD = 0.12 ng/mL) was the only congener significantly associated with increased MASLD risk and disease progression (Fig. 2a, Fig. S1, Table S1). Each doubling of PFHpA plasma levels was associated with an 80% increase in the risk of developing MASLD (OR, 1.8; 95% CI: 1.3, 2.5) (Fig. S1, Table S1). A significant dependent-response relationship between PFHpA levels and MASLD risk was observed (p_trend_< 0.0001), indicating a biological gradient across the exposure octiles (Fig. 2b). Additionally, PFHpA was significantly associated with several indicators of disease severity, including the degree of steatosis (OR=1.6, 95% CI: 1.2, 2.2 for mild steatosis and OR=1.9, 95% CI: 1.2, 2.9, moderate steatosis), fibrosis (OR =1.49, 95% CI: 1.0, 2.1), hepatocellular ballooning (OR=1.6, 95% CI: 1.0, 2.6 for few balloon cells and OR=2.8, 95% CI: 1.1, 7.6 for many balloon cells), and the NAFLD activity score (NAS) (OR=3.0, 95% CI: 1.8, 5.2) (Fig. 2c. Table S2).

### Human Study: PFHpA Affects Lipid Metabolism, Oxidative Stress, and Inflammation in Adolescents

**Figure 3.**
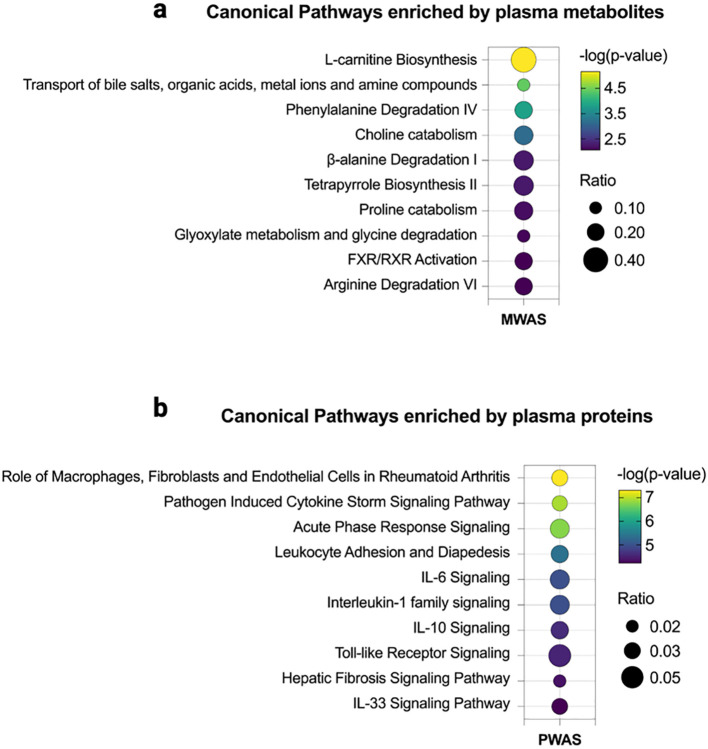
Omics-Wide Association Study in Teen-LABS. (A) Enriched pathways from the MWAS for PFHpA and metabolites from adolescents in Teen-LABS. Linear regression between PFHpA and metabolites (N=131) adjusting for age, sex, race, parental income, clinical site. (B) Enriched pathways from the PWAS for PFHpA and proteins (N=131) from adolescents in Teen-LABS. Linear regression between PFHpA and proteins (N=131) adjusting for age, sex, race, parental income, clinical site.

Using Metabolome-Wide Association Study (MWAS) and Proteome-Wide Association Study (PWAS) approaches, we identified 48 metabolites and 55 proteins that were significantly altered by PFHpA exposure (Fig. 3a and 3b, nominal p < 0.05). Ingenuity Pathway Analysis (IPA) revealed the biological pathways affected by PFHpA, with the top 10 pathways depicted in Figures 3a and 3b. The metabolites predominantly affected pathways related to amino acid metabolism, including L-carnitine biosynthesis, arginine, β-alanine, phenylalanine degradation, proline catabolism, and glyoxylate metabolism, as well as lipid metabolism, which involves the transport of bile salts, organic acids, metal ions, and amine compounds, along with FXR/RXR activation. Notably, the elevated concentrations of cholate, glucuronate, ursocholic acid, murideoxycholic acid, glycine, and glycocholic acid among the top 10 metabolites suggested a potential dysfunction in bile acid synthesis. In the plasma proteomic analysis, we used the Olink Explore^®^ cardiometabolic and inflammatory panels to identify proteins relevant to MASLD. Overall, the canonical pathways enriched by these proteins were related to chronic inflammation, cytokine signaling, and hepatic fibrosis (Fig 3b). Among the top 10 proteins from PWAS, five were directly associated with MASLD severity (CCL20^45^, CCL25^46^, ADH4^47^, IL1RN^48^, and TREM2^49^).

### *In vitro* study: PFHpA disturbs lipid metabolism in human liver spheroids.

**Figure 4.**
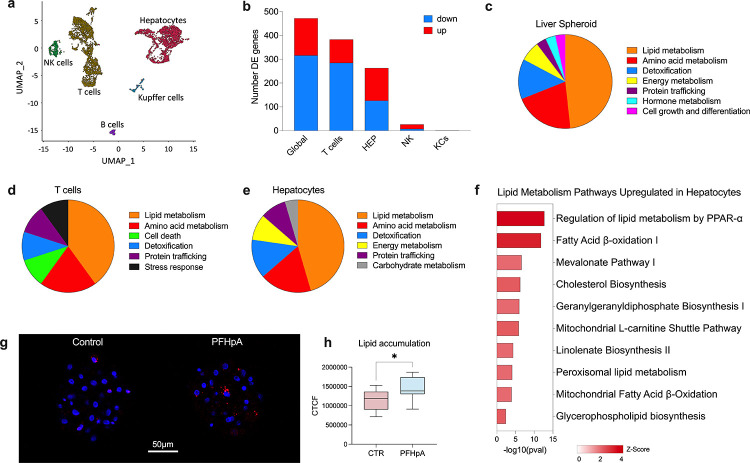
PFHpA exposure disturbs lipid metabolism in human liver spheroids. (A) Integrated UMAP of control and PFHpA-exposed spheroids. We observed the presence of hepatocytes, Kupffer cells, T cells, NK cells, endothelial cells, and B cells. (B) Differentially expressed (DE) genes detected in whole liver spheroid (global) and individual cell clusters. (C) Canonical pathways upregulated by PFHpA in whole liver spheroids. Around 48% of the pathways uploaded by PFHpA were related to lipid metabolism, while 20% were related to amino acid metabolism. Other important pathways were related to detoxification (13.8%), energy metabolism (6.9%), protein trafficking, hormone metabolism, and cell growth and differentiation. (D) Canonical pathways upregulated by PFHpA in T cells. Most of the pathways upregulated in T cells were related to lipid metabolism (40%) and amino acid metabolism (20%). Other pathways were related to cell death, detoxification, protein trafficking, and stress response. (E) Canonical pathways upregulated by PFHpA in hepatocytes. Almost 45% of the pathways upregulated in hepatocytes were related to lipid metabolism, and around 18% of the pathways were related to amino acid metabolism. Other important categories upregulated were energy metabolism, protein trafficking, and carbohydrate metabolism. (F) Upregulated pathways related to lipid metabolism in hepatocytes from spheroids exposed to PFHpA. (G) Lipid accumulation in liver spheroids. Confocal imaging of spheroids stained with Nile Red suggests an increase in lipid accumulation (red) in cells from liver spheroids exposed to PFHpA. (H) Digital quantification of confocal imaging using ImageJ confirms a significant increase in lipid accumulation due to PFHpA exposure. CTCF = Correlated Total Cell Fluorescence.

To determine the role of PFHpA in MASLD progression, we conducted an in vitro assay using human liver spheroidscomposed of primary hepatocytes and non-parenchymal cellsto PFHpA for 7 days in a low-glucose medium (Fig. 1b). Subsequent analysis using single-cell RNA sequencing (scRNA-seq) revealed significant transcriptomic alterations in the liver spheroid cells. We identified hepatocytes, T cells, Kupffer cells, and NK cells (Fig. 4a). B cells were present in minimal numbers and were therefore excluded from further analysis. Exposure to PFHpA resulted in the alteration of 472 genes in liver spheroids, with 156 upregulated and 316 downregulated genes (Excel Supplement B). Hepatocytes and T cells displayed the highest number of differentially expressed genes (DEGs), with 263 DEGs in hepatocytes (137 upregulated and 126 downregulated) and 383 DEGs in T cells (98 upregulated and 285 downregulated) (Fig. 4b). NK cells exhibited 26 DEGs (19 upregulated and seven downregulated), whereas Kupffer cells showed only the upregulation of a single gene. IPA pathway analysis highlighted the notable impact of PFHpA on liver metabolism. In the whole liver spheroids, hepatocytes, and T cells, we observed a substantial upregulation of pathways involved in lipid metabolism (48%, 45%, and 40%, respectively), amino acid metabolism (21%, 18%, and 20%, respectively), and detoxification pathways (14%, 14%, and 10%, respectively), indicating a significant disturbance induced by PFHpA exposure (Fig. 4c–e). Additionally, PFHpA exposure led to activation of peroxisome proliferator-activated receptor alpha (PPAR-α) and enhanced fatty acid oxidation in human hepatocytes (Fig. 4f). Most pathways related to hepatocyte lipid metabolism are associated with lipid anabolism (lipogenesis), with upregulation of several lipid biosynthesis pathways, including cholesterol. This was corroborated by Nile Red imaging, which revealed a significant increase in lipid accumulation in PFHpA-exposed liver spheroids compared to that in controls (p=0.01) (Fig. 4g and 4h). Although PFHpA exposure also affected lipid metabolism in T cells, the upregulated pathways were primarily associated with nuclear hormone receptor activation and did not directly affect lipid biosynthesis, unlike in hepatocytes (Fig. S2). These findings emphasize the critical role of PFHpA in disrupting lipid metabolism, particularly in hepatocytes, and suggest the potential contribution of PFHpA to MASLD pathogenesis.

### Unveiling Biomarker Signatures for PFHpA-induced MASLD through Multi-omics Integration

**Figure 5.**
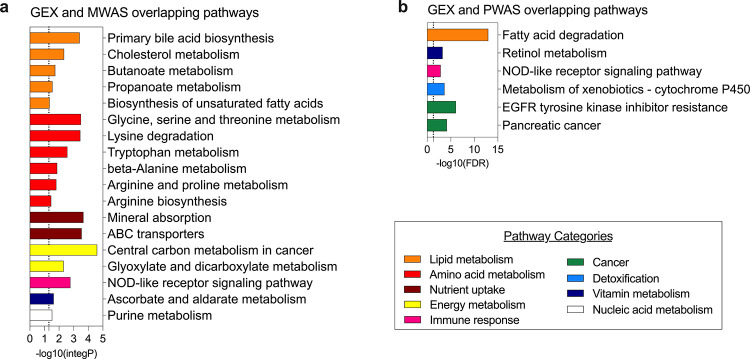
Integration of in vitro and in vivo datasets for identification of proteomic and metabolomic signatures. (A) Pathways commonly found in GEX (in vitro) and MWAS (in vivo) datasets. The shown pathways are composed of genes and metabolites. The majority of the pathways are related to lipid metabolism and amino acid metabolism. (B) Pathways commonly found in GEX (in vitro) and PWAS (in vivo) datasets. The shown pathways are composed of genes and proteins. The most significant pathway is related to lipid metabolism (fatty acid degradation [-log10(FDR)= 12.97].

We employed the OmicsNet 2.0, platform to integrate data from human and in vitro studies to identify plasma biomarkers associated with PFHpA-induced MASLD. Our multi-omics analysis combined 156 upregulated genes (GEX) from in vitro experiments with 42 metabolites (MWAS) and 28 proteins (PWAS) found at higher concentrations in the plasma of human subjects (Fig. 1c). We identified a metabolomic signature for PFHpA-induced MASLD that included 19 metabolites involved in lipid and amino acid metabolism (Fig. 5a, Table S2). Additionally, a proteomic signature was defined consisting of six proteins linked to lipid degradation and immune response pathways (Fig. 5b, Table S3).

**Figure 6.**
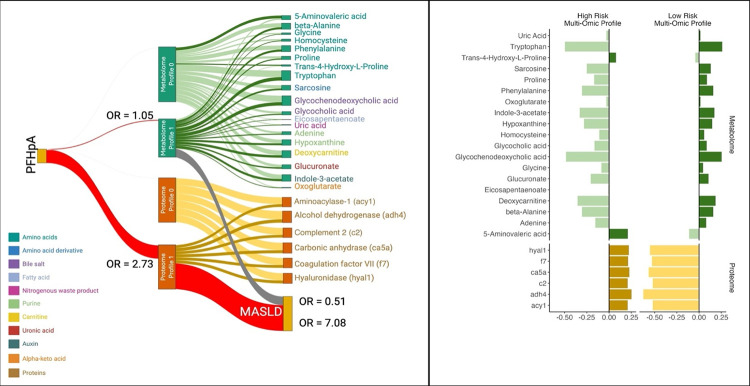
Multi-omics integration of PFHpA, proteomics, and metabolomics to determine clusters of individuals at high risk for MASLD (N=131). We identified two distinct multi-omic risk profiles associated with high PFHpA exposure and higher odds of MASLD. The first omic risk profile showed an association between high PFHpA levels and increased proteins. MASLD was 7 times more likely in this PFHpA-protein risk profile. The second omic risk profile showed an association between high PFHpA and altered levels of amino acids and lipids metabolites. (A) Shows the association of PIPS from unsupervised LUCID and MASLD (No, Yes), N= 131. The model includes the two layers in the same model. REF = NO MASLD, model adjusted for study site, age, sex, race, parental income. The reference cluster for the proteins is the high-risk cluster and for the metabolites the reference cluster is the low-risk cluster. Results are shown as log2 of PFHpA exposure and therefore interpreted as per doubling of PFHpA exposure. (B) Demonstrates the high and low risk for MASLD groups of individuals by the clusters of metabolites and proteins. Results are shown as log2 of PFHpA exposure and therefore interpreted as per doubling of PFHpA exposure.

To further analyze the relationship between PFHpA exposure, multi-omics signatures, and MASLD risk, we utilized a Latent Unknown Clustering with Integrated Data (LUCID) model^43^. This model grouped individuals based on similarities in PFHpA exposure, proteome and metabolome signatures, and disease outcomes, focusing on disease risk rather than on stratification. Fig 6a illustrates the associations between PFHpA and two latent clusters derived from each omic layer (metabolome and proteome) that characterize the low and high MASLD risk groups of individuals. PFHpA was more strongly associated with the high-risk MASLD cluster characterized by specific proteins (OR = 2.73) than with the low-risk cluster characterized by metabolites (OR = 1.05). Specifically, individuals in proteome profile 1 had significantly higher odds of MASLD (OR = 7.08) than those in proteome profile 0, while individuals in metabolome profile 1 had lower odds of MASLD (OR = 0.51) than those in metabolome profile 0. This analysis identified metabolome profile 0 and proteome profile 1 as high-risk multiomic profiles for MASLD.

Fig. 6b illustrates the differentiation between the high- and low-risk groups for MASLD based on clusters of metabolites and proteins. Key metabolites, including tryptophan, glycochenodeoxycholic acid, and deoxycarnitine, were identified as significant features that distinguished these risk groups. The combined presence of high concentrations of the metabolites Trans-4-Hydroxy-L-Proline and 5-Aminovaleric acid, aminoacylase 1 (acy1), alcohol dehydrogenase 4 (adh4), complement component 2 (c2), carbonic anhydrase 5A (ca5a), coagulation factor VII (f7), and hyaluronidase 1 (hyal1) were indicative of a higher risk MASLD group. Notably, the proteins hyal1, f7, ca5a, c2, adh4, and acy1 exhibited similar scaled values ranging from 0.21 to 0.25 for the high-risk group, while the values for low-risk group ranged from −0.63 to −0.52.

## Discussion

In this study, we integrated human epidemiological data with in vitro experimental findings to clarify the impact of perfluoroheptanoic acid (PFHpA), a newer PFAS substitute and minor degradation product of long-chain PFAS^50–53^ that accumulates in high concentrations in the liver^21^—, on MASLD risk and progression. In our analysis of the Teen-LABS cohort, we observed that doubling of PFHpA levels was linked to a 68% increase in MASLD risk. Integrative analysis revealed overlapping dysregulated pathways in both human and spheroid models, particularly those related to innate immunity, inflammation, and lipid metabolism. Our results identified a proteome profile with approximately 600% higher odds of having MASLD, whereas a distinct metabolome profile was associated with a 49% reduction in the odds of MASLD. These findings indicate specific multi-omic profiles as high-risk factors for MASLD and underscore the crucial role of protein dysregulation in disease pathogenesis. This translational framework, which integrates findings from in vitro spheroid studies with human data, can be applied to future research aimed at uncovering the molecular mechanisms of PFAS-induced liver disease and guiding the development of targeted prevention and treatment strategies for MASLD.

Among the eight PFAS congeners included in this study, only PFHpA was found to be associated with MASLD. We were particularly interested in PFHpA as it is understudied in the literature, and we found associations with MASLD (y/n) and MASLD disease severity (no disease/MASLD, MASH), and we observed a concentration-dependent relationship between PFHpA and MASLD. Notably, David et al. observed similar findings; PFHpA was the only association observed and was found to be associated with MASLD severity, including advanced steatosis and fibrosis^53^. The PFHpA-associated perturbed canonical pathways among Teen-LABS participants indicate the role of innate immunity and inflammatory markers, and when we integrate the human study with the spheroid study, we see the importance of inflammation and dysregulation of lipid-related pathways in disease progression. The PWAS analysis in Teen-LABS highlights the dysregulation of pathways related to innate immunity by PFHpA, showing enrichment of inflammatory cytokines including Interleukin-6 (IL-6), Interleukin-1 (IL-1), Interleukin-10 (IL-10), Interleukin-33 (IL-33), and Toll-like receptor signaling pathways. In addition to promoting inflammation, these pathways have important metabolic effects on lipid metabolism^54–58^. Activation of the innate immune system plays a crucial role in initiating and intensifying liver inflammation in NAFLD/NASH^59^. Multi-omics integration using LUCID revealed that proteins drive a high-risk MASLD profile. These results may be particular to our cohort as it is comprised of obese youth. While we do see that nearly 60% of the cohort has been diagnosed with MASLD, the large majority of participants had Grade 1 steatosis reflecting an earlier stage disease^60^. The LUCID results highlighted the role of chronic inflammation in the etiology of PFHpA exposure and MASLD. Studies have shown that several proteins included in the high-risk profile play a role in the development of fibrosis, a late stage of liver disease progression seen in MASLD, including carbonic anhydrase^61,62^, coagulation factor VII^63–65^, and hyaluronidase^66–69^. In particular, hyaluronidase, an enzyme that breaks down hyaluric acid, is relevant, as increased hyaluric acid can contribute to the degradation of the extracellular matrix, which may affect liver fibrosis progression^66–69^. Although a direct link between complement 2 (C2) and MASLD has not yet been firmly established, the involvement of the complement system in inflammation and immune responses suggests that C2 may play a role in disease’s progression^70–72^. Chronic inflammation and immune-mediated liver injury, both influenced by complement activation, are key aspects of MASLD pathogenesis^71–74^. Further research is needed to clarify the role of these mechanisms in MASLD and determine whether they could serve as therapeutic targets or biomarkers.

To delve deeper into the molecular mechanisms underlying PFHpA-associated MASLD, we conducted *in vitro* experiments to examine how PFHpA affects liver metabolism, using a 3D human liver spheroid co-culture model. Our study used single-cell transcriptomics to evaluate the impact of PFHpA on hepatic cell populations in liver spheroids. These findings reveal that PFHpA primarily affects lipid metabolism, leading to a notable increase in anabolic events in human primary hepatocytes, with significant hepatic lipid accumulation after PFHpA exposure. Among the pathways involved in lipid metabolism in hepatocytes, the ‘Regulation of lipid metabolism by PPAR-α’ and ‘Fatty Acid β-oxidation I’ pathways showed the strongest activation. This suggests that PFHpA promotes hepatocyte lipogenesis, likely through peroxisome proliferator-activated receptor (PPAR)-α signaling. PPARs are members of the nuclear hormone receptor superfamily that act as ligand-activated transcription factors^75^. In the liver, PPAR-*α* plays a crucial role in regulating fatty acid oxidation and lipid and lipoprotein metabolism^76^. Previous research has shown that various PFAS, including PFHpA, can activate PPAR-α in cell lines and animal models^77–79^. Recently, Yang et al. proposed that the hepatic lipid metabolism disruption caused by PFOA and PFOS depends on the PPAR-α/ACOX1 axis^80^. Our *in vitro* analysis indicated that this pathway is disrupted in hepatocytes, but not in immune cells. We found that ACOX1 expression was upregulated and integrated the pathway ‘Regulation of lipid metabolism by PPAR-α’ in hepatocytes from spheroids exposed to PFHpA. In contrast, while PPAR-α signaling was also upregulated in T cells exposed to PFHpA, ACOX1 was not differentially expressed and no lipid biosynthesis pathways were detected (Excel Supplement B). Our findings indicate that PFHpA alter signaling of PPAR-฀/ACOX1 axis in hepatocytes but not T cells, to instigate abnormal hepatic lipid metabolism in humans.

In addition to establishing a clear connection between PFHpA exposure and MASLD in humans, our study elucidated the intricate molecular mechanisms by which PFHpA affects liver metabolism. Furthermore, Teen-LABS comprises adolescents; therefore, the results may not be generalizable to an older population. However, our findings are strengthened by the integration of results from a spheroid experimental study and an epidemiological study. Moreover, we developed a translational research framework integrating epidemiological and bench science research that allowed us to identify individuals at a high risk of MASLD due to PFHpA exposure. Our framework not only advances the current knowledge on the impact of PFASs on chronic liver diseases, but also provides critical information for future policies aimed at mitigating the detrimental impact of PFASs on human health. Finally, our findings offer potential new targets for MASLD treatment strategies by determining the specific molecular targets implicated in PFAS-induced liver disease. In conclusion, we used a translational framework to show that PFHpA is associated with MASLD and that the underlying mechanisms are related to innate immunity, inflammation, and lipid dysregulation.

## Methods

### Study population.

This study was based on data from the Teen-LABS (Longitudinal Assessment of Bariatric Surgery) study (ClinicalTrials.gov number, NCT00465829), a prospective, multicenter, observational study of adolescents (≤19 years of age) who underwent bariatric surgery between 2007 and 2012. Participants were enrolled at five clinical centers in the United States: Cincinnati Children’s Hospital Medical Center (Cincinnati, Ohio), Nationwide Children’s Hospital (Columbus, Ohio), the University of Pittsburgh Medical Center (Pittsburgh, Ohio), Texas Children’s Hospital (Houston, Texas), and the Children’s Hospital of Alabama (Birmingham, Alabama)^24^. Study inclusion criteria were (1) adolescents up to 19 years of age, (2) adolescents approved for bariatric surgery, and (3) agreement to participate in the Teen-LABS study, demonstrated through the signing of Informed Consent/Assent^24^. The Teen-LABS steering committee, which included a site principal investigator from each participating center, collaborated with the data coordinating center and project scientists from the National Institute of Diabetes and Kidney Disease (NIH-NIDDK) to design and implement the study^24^. All bariatric procedures were performed by surgeons who were specifically trained in study data collection (Teen-LABS-certified surgeons)^24–27^. The present study included 136 participants whose plasma was collected at the time of surgery. The study protocol, assent/consent forms, and data and safety monitoring plans were approved by the Institutional Review Boards of each institution, and by the independent data and safety monitoring board prior to study initiation^24^. Written informed consent or assent, as appropriate for age, was obtained from all parents/guardians and adolescents^24^. This study was approved by the University of Southern California Review Board.

### Data collection.

Standardized methods for data collection have been described previously^24–27^. Fasting blood samples were obtained preoperatively. Liver histology and liver biopsy methodology have been previously detailed^27^, however, liver biopsies were obtained using the core needle technique after anesthesia induction and before performing the bariatric surgery procedure. Owing to the observational study design and lack of published consensus on whether intraoperative liver biopsies should be the standard of care at the time of bariatric surgery, the decision to perform a liver biopsy was deferred to the surgical teams at each site. Accordingly, 99% of all biopsies were performed at sites where intraoperative biopsy is the standard of care. Liver biopsy specimens were stained with hematoxylin-eosin and Masson’s trichrome stains, reviewed, and scored centrally by an experienced hepatopathologist using the validated MASH Clinical Research Network scoring system^28^. MASLD was defined based on the histopathological diagnosis. Detailed descriptions of study methods, comorbidity and other data definitions, case report forms, and laboratory testing have been included in previous publications^24–26^. For this analysis, covariates, including participants’ age, sex assigned at birth, race, and parents’ income, were obtained at the time of surgery by trained study personnel^24,25^. The collected data were maintained in a central database by the data-coordinating center.

### Plasma-PFAS Laboratory Analysis.

The samples were transported on dry ice with temperature logging by a World Courier (AmerisourceBergen Corporation, Conshohocken, PA) and stored at −80°C until analysis. The samples were analyzed by online solid-phase extraction followed by LC-MS/MS, as previously described. The limit of detection (LOD) as 0.03 ng/mL for all reported compounds. Values below the LOD were imputed as ½ LOD. The batch imprecision for the quality control samples was less than 6% for all measured compounds. Plasma concentrations of PFAS measured in Teen-LABS participants have been previously published^21^.

### Plasma Metabolomics.

Untargeted plasma metabolomics was performed on plasma samples collected at the time of bariatric surgery. Liquid chromatography coupled with high-resolution mass spectrometry (LC-HRMS) was used as described by Liu et al.,^31^ with dual-column and dual-polarity approaches and both positive and negative electrospray ionization. This resulted in four analytical configurations: reverse-phase (C18)-positive, C18 negative, hydrophilic interaction (HILIC) positive, and HILIC negative. Unique features were identified using mass-to-charge ratio (m/z), retention time, and peak intensity. Features were adjusted for batch variation^32^ and excluded if they were detected in < 20% of the samples or if there was a > 30% coefficient of variability of the quality control samples after batch correction. After processing, there were 3,716 features from the C18 negative mode, 5,069 from the C18 positive mode, 7,444 from the HILIC negative mode, and 6,944 from the HILIC positive mode for a total of 23,173 features included in the analyses. The raw intensity values from LC-HRMS were scaled to a standard normal distribution and log_2_ transformed. The details of the analytical process have been described previously^33^. Confirmed annotations with a confidence level of 1 were available for 358 metabolomic features^34^. Metabolites were identified by comparing them with authentic chemical standards under identical analytical conditions, and peaks were matched to annotations using m/z and retention time. In instances where multiple annotations were possible because more than one molecule had retention times within the allowable error, the annotation with the closest retention time to the known standard was chosen. The measured m/z and retention times, theoretical m/z and retention times, adducts, possible annotations, and additional analytical details are listed in Excel Supplement A.

### Plasma Proteomics.

Proteins were measured in fasting plasma samples using the proximity extension array (PEA) method from the Olink Explore 384 Cardiometabolic panel and Olink Explore 384 Inflammation panel^35^. These panels measure the relative abundance of 731 proteins, reported as normalized protein expression (NPX) levels after log_2_ transformation^36^. After excluding proteins with over 50% of observations below the limit of detection (LOD) and duplicate proteins, 702 proteins were retained from the initial 731 offered after processing.

### Liver spheroid assay.

We used a 3D InSight^™^ Human Liver Model (MT-02-302-04, InSphero Inc.) to test the impact of PFHpA on human liver metabolism. This model is composed of human primary hepatocytes from 10 donors (five males and five females) and non-parenchymal cells from one donor. A PFHpA (CAS#375-85-9, Sigma-Aldrich, cat# 342041) stock solution was prepared in dimethyl sulfoxide (DMSO, Sigma-Aldrich). The final working solution was diluted in lean spheroid medium (CS-07-305B-01, InSphero Inc.) to a final non-cytotoxic concentration of 20μM μM PFHpA and 0.1% DMSO^37^. Liver spheroids were continuously exposed to PFHpA for 7 days, and the culture medium was replaced every 2–3 days with media containing freshly diluted PFHpA. For the control, liver spheroids were exposed to 0.1% DMSO diluted in lean spheroid media and cultured for 7 days, following the same regimen of media replacement. Spheroids were cultured in a 96-well format, with a single spheroid per well, under sterile conditions and incubated at 37 °C and 5% CO_2_ following the manufacturer’s instructions. We used 96 spheroids per condition.

### Lipid accumulation assay and analysis.

After 7 days of treatment, the spheroids were fixed with 4% paraformaldehyde in phosphate-buffered saline (PBS) for 1h, permeabilized with 0.2% Triton-X100 in PBS for 30 min, and blocked with 1% bovine serum albumin (BSA) in PBS for 1h at room temperature. Spheroids were then stained with Nile Red (1μg/mL) and DAPI (2μg/mL) in 1% BSA/PBS for 30 min and washed 3x with PBS. Spheroids were mounted with Prolong Gold Mounting Medium (Invitrogen) and images were acquired using a Leica TCS SP8 confocal microscope. Lipid accumulation was quantified using ImageJ^38^.

### Single-cell RNA library preparation, sequencing, and data analysis.

Spheroids were dissociated using 0.25% trypsin for 15 min and dead cells were removed using a Dead Cell Removal Kit (Miltenyi Biotech). Viable cells were partitioned with the Chromium Next GEM Single Cell 3ʹ Kit (10X Genomics). Libraries were sequenced on a USC Molecular Genomics Core using the Illumina platform. Raw data were processed using the Cell Ranger count pipeline (10X Genomics) with low-quality cell removal according to sample-specific quality control (QC) using the R package Seurat^39^. Comparison between PFHpA-treated and control samples was performed by integrating samples into a unified dataset using the SCTransform integration workflow implemented in Seurat^40^. Cell annotation was based on the expression of known cell type marker genes. Differentially expressed genes between clusters from treatment groups were detected using the Wilcoxon Rank Sum test in the Seurat FindMarkers, and genes with a Bonferroni adjusted p-value < 0.1 were considered significant. Biological data interpretation was performed using Ingenuity Pathways Analysis (IPA)^41^.

### Multi-omics data integration.

We performed knowledge-driven integration of the differentially expressed genes obtained from the transcriptomic PFHpA/liver spheroids in vitro assay (GEX), and the plasma proteome wide association study (PWAS) and metabolome wide association study (MWAS) data obtained from the Teen-LABS cohort using the online platform OmicsNet 2.0 (www.omicsnet.ca)^42^. Briefly, OmicsNet identified the significant canonical pathways in each dataset and separately overlapped the results from GEX, PWAS, GEX, and MWAS. We then identified the proteins and metabolites in the overlapping pathways and used them as omics signatures for further analysis using latent unknown clustering integrating multi-omics data (LUCID).

### Statistical Analysis

#### Plasma-PFHpA and MASLD:

We first evaluated the associations of eight PFAS measured in plasma with MASLD (yes/no) using logistic regression and controlling for multiple comparisons using Bonferroni correction. Plasma plasma-PFAS concentrations were log2 transformed. PFHpA was the only congener found to be associated with MASLD in our study; therefore, we focused on subsequent analyses to further explore the association between plasma PFHpA and MASLD and its features using either multinomial logistic regression models or logistic regression models, based on the number of categories in each outcome. The outcomes of interest included the progression of MASLD (No MASLD, MASLD not MASH, MASH, multinomial regression), hepatocellular ballooning (none, few, and many; multinomial logistic regression), grade of steatosis (none, 5–33%, 34–67%; multinomial logistic regression), fibrosis (none, present; logistic regression), and MAS activity score (none, 1, 2, ≥3; multinomial logistic regression). For all models, we adjusted for participants’ age, race, sex assigned at birth, parents’ annual income, and site of the medical center. To test the concentration-dependent relationship between plasma-PFHpA and MASLD, we used quantiles of PFHpA exposure with a continuous exposure value to determine the trend P value across quantiles depicting a readily interpretable dose-response relationship.

#### Metabolome-wide association study (MWAS) and Proteome-wide association study (PWAS—linking PFHpA exposure with disease pathways:

To perform the metabolome- and proteome-wide analysis study analysis, we included confirmed annotations with confidence level 1 metabolomic features^34^ and NPX levels as the dependent variables and the log2-transformed PFHpA concentration as the independent variable in a multiple linear regression model. In both the MWAS and PWAS models, we adjusted for participants’ age, race, sex assigned at birth, parents’ annual income, and site of medical centers to control for potential confounding. As we were not primarily interested in strict feature (protein or metabolite) identification, we conducted an enrichment analysis for features that were altered by PFHpA exposure (nominal p < 0.05) using canonical pathways and diseases and biofunctions curated from Qiagen Knowledge Base using QIAGEN Ingenuity Pathway Analysis (IPA, Qiagen Inc.).

#### Multi-omics integration-Latent Unknown Clustering by Integrating multi-omics Data (LUCID):

Latent Unknown Clustering by Integrating multi-omics Data (LUCID) is a novel quasi-mediation analysis approach of multi-omics data that estimates the joint associations between the environmental exposure E (PFHpA), the multi-omics data Z (19 metabolites and 6 proteins that were identified through prior pre-screening procedures), and the outcome Y (MASLD) if supervised via the latent cluster variable X. The Expectation-Maximization (EM) algorithm was implemented to iteratively estimate X and update the parameters until convergence. For an unsupervised LUCID model, the parameters of interest include (1) β, representing PFHpA-to-cluster associations; (2) μ, representing the cluster-specific means of omics features; and (3) the individual-level inclusion probability (IP) for each latent cluster. In the unsupervised LUCID approach, X integrates information from both E and Z, effectively delineate distinct risk profiles among the subjects. The original LUCID framework was initially proposed for the early integration of multi-omics data, entailing concatenation of all omics layers into a single matrix. Detailed descriptions of the original LUCID have been previously introduced^43^. As an extension of the original LUCID, LUCID in parallel utilizes an intermediate integration strategy of multi-omics data to estimate separate latent clusters X within each omic layer by assuming no correlations across different omics layers^44^. In our study, there were two layers of multi-omics data, Z, metabolites, and proteins, resulting in two individually estimated latent cluster variables, X_metabolome_ and X_proteome_. We fitted the optimal unsupervised LUCID in parallel model using PFHpA as E and pre-selected 19 metabolites and 6 proteins as Z. Based on model selection procedures using Bayesian information criterion (BIC), the number of latent clusters per omic layer of the optimal model was chosen to be 2. We extracted the IPs to X_metabolome_ and X_proteome_ (IP_metabolome_ and IP_proteome_) from the converged optimal LUCID in the parallel model. IP_metabolome_ and IP_proteome_ are continuous probabilities indicating the likelihood of being included in each level of the cluster X_metabolome_ and X_proteome_, respectively. These probabilities were determined by the subjects’ exposure levels and the presence of metabolites and proteins, respectively. In follow-up analyses to explore how IP_metabolome_ and IP_proteome_ were associated with the outcome of interest, MASLD, we fitted a logistic regression model using IP_metabolome_ and IP_proteome_ as predictors and MASLD as the binary response variable, while adjusting for covariates, including age, sex, race, parents’ income, and study site.

## Data Availability

Raw and processed scRNA-seq datasets were deposited in the NCBI GEO database under accession number GSE253186. Access to data can be achieved by requesting the Teen-LABS steering committee and NIH-NIDDK biobank.

## Impact:

The rising prevalence of metabolic dysfunction-associated steatotic liver disease (MASLD), particularly in children, emphasizes the urgent need to identify modifiable risk factors to control disease progression. This study provides novel insights into the role of perfluoroheptanoic acid (PFHpA), an unregulated polyfluoroalkyl substance (PFAS) congener, in MASLD development, showing that elevated PFHpA levels are significantly associated with increased MASLD risk. These findings are crucial for researchers, clinicians, and public health officials aiming to inform policy and understand environmental factors contributing to MASLD, especially vulnerable pediatric populations. By identifying PFAS-induced molecular pathways linked to MASLD, this work may inform precision health strategies and targeted interventions for prevention and treatment.

## List of Abbreviations:

DEGs: differentially expressed genes
GEX: gene expression
IPA: Ingenuity Pathway Analysis
LUCID: latent unknown clustering with integrated data
MASH: steatohepatitis
MASLD: metabolic dysfunction-associated steatotic liver disease
MWAS: Metabolome-Wide Association Study
PFAS: Per- and polyfluoroalkyl substances
PFHpA: perfluoroheptanoic acid
PFOA: perfluorooctanoate
PFOS: perfluorooctane sulfonate
PPAR-α: peroxisome proliferator-activated receptor alpha
PWAS: Proteome-Wide Association Study
